# Calcium Signalling and Liver Regeneration

**DOI:** 10.1155/2012/630670

**Published:** 2012-10-16

**Authors:** Isabelle Garcin, Thierry Tordjmann

**Affiliations:** ^1^INSERM U.757, Université Paris Sud, Bât. 443, 91405 Orsay, France; ^2^Université Paris Sud, Bât. 443, 91405 Orsay, France

## Abstract

After partial hepatectomy (PH) the initial mass of the organ is restored through a complex network of cellular interactions that orchestrate both proliferative and hepatoprotective signalling cascades. Among agonists involved in this network many of them drive Ca^2+^ movements. During liver regeneration in the rat, hepatocyte cytosolic Ca^2+^ signalling has been shown on the one hand to be deeply remodelled and on the other hand to enhance progression of hepatocytes through the cell cycle. Mechanisms through which cytosolic Ca^2+^ signals impact on hepatocyte cell cycle early after PH are not completely understood, but at least they include regulation of immediate early gene transcription and ERK and CREB phosphorylation. In addition to cytosolic Ca^2+^, there is also evidence that mitochondrial Ca^2+^ and also nuclear Ca^2+^ may be critical for the regulation of liver regeneration. Finally, Ca^2+^ movements in hepatocytes, and possibly in other liver cells, not only impact hepatocyte progression in the cell cycle but more generally may regulate cellular homeostasis after PH.

## 1. Introduction

After partial hepatic destruction in experimental or clinical context, the initial mass of the organ is restored through compensatory growth of the remnant liver. A complex and yet incompletely elucidated network of cellular interactions (including paracrine, autocrine, endocrine, or nervous pathways) orchestrates the regulation of regeneration, through both proliferative and hepatoprotective signalling cascades [1]. A number of agonists constituting this network drive intracellular Ca^2+^ movements, in particular through the formation of inositol 1,4,5 trisphosphate which binds on its receptor in the membrane of the endoplasmic reticulum, and release the Ca^2+^ stored in this organelle. Such agonists include some of the main comitogenic—as noradrenalin [2], arginine vasopressin (AVP) [3], and adenosine triphosphate (ATP) [4]—and mitogenic factors, as epidermal growth factor (EGF), hepatocyte growth factor (HGF) [5], and insulin [6]. The resulting increase in ionized cytosolic calcium concentration generally consists of a regular succession of Ca^2+^ peaks (oscillations) [7] that can be transmitted to other cells (intercellular calcium waves) which mechanisms and functions are not fully known [8, 9]. The impact of calcium signalling on liver regeneration has, however, only been scarcely studied.

## 2. Hepatocyte Ca^2+^ Signalling: Mechanisms and Functions

The first calcium oscillations were reported in hepatocytes [10], and numerous studies, both experimental and theoretical, have been conducted after that to decipher, in the hepatocyte, the machinery by which agonists generated cytosolic calcium signals. However, the functions of hepatocyte calcium signalling remain far less understood.

In hepatocytes, as in most nonexcitable cells, Ca^2+^ oscillations originate from the periodic opening of Ca^2+^ channels located in the ER membrane, following activation of the phosphoinositide cascade. The binding of an agonist to a membrane-bound receptor activates the G*α*-subunit of a G-protein complex coupled to the receptor. This activated G protein in turn stimulates phospholipase C (PLC) activity. The latter enzyme catalyzes the hydrolysis of the membrane-bound phosphatidyl-inositol bisphosphate (PIP_2_) into diacyl-glycerol and inositol trisphosphate (InsP_3_). Ca^2+^ release from the internal stores is ensured by the InsP_3_R, an homotetramer that can bind up to 4 InsP_3_ molecules, forming a Ca^2+^ channel which equilibrium open probability presents a bell-shaped dependence on cytosolic Ca^2+^ [11]. The decrease of [Ca^2+^]_i_ in the cytosol is due to the activity of the Ca^2+^ ATPases (SERCA pumps), which actively transports Ca^2+^ from the cytosol into the ER. Ca^2+^-regulated InsP_3_Rs and Ca^2+^ ATPases are together sufficient to generate Ca^2+^ oscillations [12]. In most cases, hormone-induced Ca^2+^ oscillations in hepatocytes take the form of repetitive, sharp spikes sometimes preceded by a slower, pacemaker-like elevation in the cytosolic Ca^2+^ concentration. These periodic increases in the level of free Ca^2+^ in the cytosol from about 0.1 *μ*M up to 1 *μ*M have been observed in hepatocytes in response to stimulation by a large number of agonists such as noradrenalin, vasopressin, phenylephrin, angiotensin II, adenosine triphosphate (ATP), histamine, and thrombin, the shape of the oscillations being agonist dependent [7]. The oscillation frequency increases with the agonist concentration, a phenomenon known as “frequency encoding”, and is affected by external [Ca^2+^]—and thus by the rate of Ca^2+^ entry into the cell through plasma membrane Ca^2+^ channels. Intracellular Ca^2+^ waves do not result from a simple diffusion of Ca^2+^ itself—which is quickly buffered—but from the spreading of InsP_3_—which is more soluble in the cytosol—that mobilizes Ca^2+^ from storage compartments throughout the cell. A particular spatial pattern of subcellular InsP_3_R distribution was reported to support the direction of intracellular Ca^2+^ waves, starting from the canalicular region containing the most abundant and affine InsP_3_R isoform (type II InsP_3_R) and spreading toward the other regions of the cytosol, less sensitive to InsP_3_ [13]. As in many other cell types, intracellular movements of Ca^2+^ in hepatocytes, induced by hormones and neurotransmitters, may be propagated from cell to cell. Our group demonstrated in multicellular rat hepatocyte systems (couplets and triplets) that agonists such as vasopressin or noradrenalin induce tightly coordinated and sequentially ordered intracellular Ca^2+^ increases [8, 14–16]. Such signals were also observed in the intact perfused liver in which vasopressin elicits waves of [Ca^2+^]_i_ increase running along hepatocyte plates across the lobules [17–19]. We demonstrated that unidirectional Ca^2+^ waves resulted from a gradually decreasing cellular sensitivity to hormonal stimuli from the first to the last responding cell, and that this cell to cell heterogeneity was due to a lobular gradual distribution of hormonal receptors density [8]. Moreover, InsP_3_ has been shown to flow through gap junctions and thereby coordinate Ca^2+^ spiking among adjacent hepatocytes [20]. Such a configuration in which the most responsive hepatocytes drive the response of the less sensitive cells is similar to the cell to cell triggering of cardiac pacemaker cells [8, 21, 22].

In general terms, Ca^2+^ oscillations in hepatocytes optimize the effect of hormonal stimulation, thanks to enzymes decoding [23] their frequency [24, 25], as proposed for glycogen metabolism in hepatocytes [26]. Since the early data showing that production of glucose by the liver was at least in part mediated by hormone-induced intracellular Ca^2+^ increases [27], most recent studies have deciphered the molecular mechanisms linking intracellular Ca^2+^ to glucose metabolism in hepatocytes [28–30]. In particular, the serine-threonine kinase “calcium calmodulin-dependent kinase II” (CaMKII), a major mediator of Ca^2+^ signalling in different cell types, has been found to play essential roles in the regulation of glycogenolysis and gluconeogenesis in hepatocytes, not only during physiological fasting, but also in the pathophysiological setting of obesity [28, 30]. It is moreover well established that Ca^2+^ oscillations in hepatocytes coordinate intramitochondrial ATP synthesis with cellular energy demand, maintaining cell homeostasis and viability [31]. It has also been demonstrated that [32] the temporal pattern of calcium signals was of major impact as to the expression of transcription factors in lymphocytes, but this aspect has never been investigated in hepatocytes. Many events related to bile secretion are also regulated by cytosolic Ca^2+^, such as vesicular trafficking and canalicular exocytosis of bile acid transporters [33, 34], permeability of tight junctions [35], or canalicular contraction [9, 36]. Intracellular calcium waves, as described above, starting from the canaliculus to the basolateral poles may have physiological impact on secretion, as it has been shown in pancreatic acinar cells [37], although direct evidence in hepatocytes is lacking. Moreover, interhepatocyte calcium waves have been reported to support canalicular peristaltism and thereby to regulate bile flow, in the normal and regenerating rat liver [3, 38]. As emphasized in the following, all these Ca^2+^-regulated physiological processes may impact the course of liver regeneration.

## 3. Intracellular Calcium and Hepatocyte ****Proliferation: Liver Regeneration

It is well established that intracellular Ca^2+^ is crucial for tissue homeostasis through regulation of cell cycle and apoptosis [7]. In particular, intracellular calcium has been reported to regulate cell proliferation at multiple steps of the cell cycle, from immediate early genes activation, toward G1-S and G2-M transitions, as well as during mitosis [39]. Pioneer studies have shown that extracellular calcium was crucial for liver regeneration [40]. Also, modifications of intracellular calcium homeostasis during liver regeneration have been reported, concerning Ca^2+^-binding proteins [41], membrane Ca^2+^-ATPases [42], or the InsP_3_ receptor [43, 44]. It has been also suggested that the alteration of the InsP_3_ and Ca^2+^mobilisation pathway could alter liver regeneration in the rat [45]. Subsequently, it has been shown in nonhepatocytic cell lines that the spatiotemporal organisation of Ca^2+^ signals was determined for the activation of transcription factors like CREB, NF-*κ*B, or NF-AT, and for immediate early genes like c-fos or c-jun [46–48]. It is also well established that the activation of the RAS pathway is controlled by [Ca^2+^]_i_ oscillations [49]. Moreover, intra-nuclear calcium signals, which have been well documented [50], have a major impact on gene transcription [51–53] and can result either from the diffusion of cytosolic calcium to the nucleus, or from an InsP_3_ -mediated calcium release in the nucleus itself (see below) [6, 54]. Finally, our previous work demonstrated that hepatocyte calcium signalling was deeply remodelled during liver regeneration in the rat, contributing to the regulation of bile flow and cell proliferation [3, 55].

In a recent study, we examined the physiological involvement of cytosolic calcium during liver regeneration in the rat [56]. We interfered with calcium signalling before PH by expressing parvalbumin (PV) in the liver, a calcium-binding protein expressed in muscle cells and neurons but absent from the liver [57], using adenoviruses coding for PV targeted to the cytosol, to selectively buffer Ca^2+^ in this compartment [53, 54]. We found that expression of PV efficiently buffers agonist-induced calcium oscillations in the cytosol and inhibits primary hepatocyte proliferation *in vitro* as well as *in vivo* during liver regeneration. 

We found that immediate early gene transcription, early phosphorylation of ERK and CREB, and hepatocyte progression in the cell cycle after PH were inhibited in rats expressing cytosolic PV [56]. These data were in line with previous reports describing these pathways and genes as dependant on cytosolic and/or nuclear calcium signalling [46, 58–60]. We thus suggested that attenuated [Ca^2+^]_i_ oscillations in calcium-buffered hepatocytes resulted in impaired activation of these pathways. A potential reduction in CaM-kinase activation, as previously reported [61], or reduced ERK1/2 activation that we observed in PV-NES expressing hepatocytes may have also contributed to altered CREB phosphorylation. Since CaM-kinase II [24], as well as ERK1/2 [60] activity, is sensitive to Ca^2+^ oscillation frequency, an attractive hypothesis would be that cytosolic PV expression, by attenuating agonist-generated Ca^2+^ signals, resulted in impaired phosphorylation of CREB. 

Cytosolic calcium signalling impacts most likely the early triggering of hepatocyte progression from G0 to G1 and S phases. In line with this view, a rise in concentration—in the liver and in the plasma—is observed early after PH for several Ca^2+^-mobilizing agonists, suggesting these agonists might be involved in initiating the regeneration process. In particular, EGF and HGF elicit cytosolic Ca^2+^ oscillations in hepatocytes, the physiological impact of which has never been specifically addressed [5]. Also, extracellular ATP [4], arginine vasopressin [3], and noradrenalin [2], which are comitogenic Ca^2+^-mobilizing agonists, have been individually reported to contribute to early phases of liver regeneration. Our study thus suggested that buffering hepatocyte calcium signals, potentially generated by these agonists in the minutes after PH, result in delaying hepatocyte cell cycle progression.

There is evidence in the literature for the crucial role of mitochondrial calcium in the regulation of apoptotic processes. It is well known in particular, that mitochondrial calcium overload can be one of the pathways leading to the swelling of mitochondria and to the rupture of the outer membrane, in turn releasing proapoptotic molecules in the cytosol. Mechanisms for excessive calcium transfer to mitochondria are debated and include mainly interactions between proteins of the Bcl2 family and the InsP_3_-R. Anti-apoptotic members appeared as reducing calcium transfer from the ER to the mitochondria, whereas proapoptotic factors were reported to enhance this flux [62]. In this context, recent data suggest that mitochondrial Ca^2+^, as well as cytosolic Ca^2+^, may be critical for the regulation of liver regeneration after PH in the rat [63]. The authors suggested that buffering calcium in the mitochondria resulted in a shift in the balance between pro- and antiapoptotic factors, thereby protecting hepatocytes from apoptosis, *in vitro* in an hepatoma cell line, as well as *in vivo* in the rat liver after PH. 

## 4. Nuclear Calcium Signalling and Liver Cell Proliferation

Previous studies have established that growth factors important for liver regeneration such as HGF [54] and insulin [6] can differentially affect cytosolic and nuclear calcium in hepatocytes. It has been reported that agonist-induced calcium movements in the nucleus can schematically result from the diffusion of cytosolic calcium to the nucleus and/or from an autonomous InsP_3_ generation and calcium mobilization from local, intra, or perinuclear Ca^2+^ stores [64, 65]. Nuclear InsP_3_ may again come from the cytosol or be generated in the nucleus [6]. Indeed, the nucleus, its envelope and the nucleoplasmic reticulum, has been shown to possess several crucial molecules involved in Ca^2+^ storage, InsP_3_ production, and calcium release, therefore opening the possibility that local nuclear Ca^2+^ signals may occur independently from the cytosol [64, 65]. Although these two views may coexist in the same cells according to circumstances and cell types, it has been shown in SkHep cells that an InsP_3_-sensitive  intranuclear calcium compartment (i.e., the “nucleoplasmic reticulum”) exists [50]. PLC*β*, PIP_2_, and InsP_3_R have been found in the nucleus, allowing a local InsP_3_ production and providing the machinery necessary to generate autonomous Ca^2+^ signals [64, 65]. We also know that calcium signals in these two compartments—cytosol and nucleus—can have different effects [51, 58]. Recent works revealed that buffering calcium in the nucleus, but not in the cytosol, in a hepatoma cell line, resulted in an inhibition of cell proliferation, suggesting that nuclear Ca^2+^ was necessary for centrosome separation and cell progression through early prophase [53]. Gomes et al. further showed that the HGF receptor (c-met) can translocate (upon agonist stimulation) from the plasma membrane to the nucleus and generate an InsP_3_ production and calcium elevation in the nucleus, independently of cytosolic calcium, in a hepatoma cell line [54]. Very similarly, it was shown by the same group that insulin can induce nuclear calcium signals through a translocation of its receptor to the nucleus, in primary rat hepatocytes [6]. Importantly, the nucleoplasmic reticulum as an intranuclear calcium compartment has not been shown in primary hepatocytes, and some authors claimed that it was not essential for calcium signalling [66].

Important cellular functions are thought to be regulated by nuclear calcium signals, including nuclear pore permeability, transcription factor activity and protein kinase translocation, thereby controlling gene expression [65]. In particular, the transcriptional activity of CREB [58], NFAT [46], and DREAM [67] has been well described as dependent on nuclear Ca^2+^. Therefore, agonist-induced nuclear Ca^2+^ movements are potentially expected to impact both hepatocyte progression in the cell cycle and more generally cellular homeostasis after PH.

It is important to realize that liver regeneration, seen as a process involving the whole organ—and even the entire organism—cannot be restrained to the sole hepatocyte division. In that view, after PH, Ca^2+^ signalling may also regulate physiological processes unrelated directly to cell cycle control, although they may ultimately interfere with cell cycle progression, not only in hepatocytes but also in other liver cell types (cholangiocytes, endothelial and Kupffer cells). Whereas we do not have any direct data about the impact of Ca^2+^ signals in nonhepatocytic cells after PH, we can easily anticipate about Ca^2+^-dependent physiological processes that may be crucial in hepatocytes after PH. For example, glucose homeostasis, which regulation is critical for hepatocyte exit from quiescence [68], may depend on Ca^2+^ signals early after PH. Also, biliary homeostasis, which adaptation after PH is critical for liver regeneration [69, 70], may involve Ca^2+^-dependent regulation [3, 71]. 

**Figure 1:**
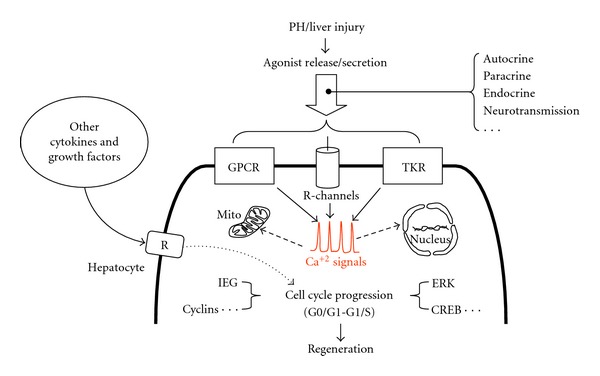
A simplified view of the impact of hepatocyte calcium signals during liver regeneration. After PH or toxic liver injury, a number of calcium mobilizing agonists are released inside or outside the liver, interacting with hepatocytes through autocrine, paracrine, and endocrine pathways. Diverse membrane receptors, either G protein coupled receptors (GPCR), tyrosine kinase receptors (TKR), or receptor channels (e.g., ionotropic purinergic receptors), can be involved in the generation of cytosolic calcium signals. These calcium movements in the cytosol can be transferred to other crucial compartments like the mitochondrion (Mito) or the nucleus, in which they could regulate respectively apoptosis and gene transcription. Previous studies have shown that cytosolic calcium signals regulate cell cycle progression from G0 to G1 and from G1 to S phases in hepatocytes after PH, at least in part through an impact on immediate early genes transcription, cyclin expression, and ERK and CREB phosphorylation.

An integrated full picture of the “liver calcium signalling”, which is obviously lacking, may improve our knowledge on the interaction network that regulates liver regeneration processes (Figure 1).
